# Effects of Late Administration of Pentoxifylline and Tocotrienols in an Image-Guided Rat Model of Localized Heart Irradiation

**DOI:** 10.1371/journal.pone.0068762

**Published:** 2013-07-22

**Authors:** Vijayalakshmi Sridharan, Preeti Tripathi, Sunil Sharma, Peter M. Corry, Eduardo G. Moros, Awantika Singh, Cesar M. Compadre, Martin Hauer-Jensen, Marjan Boerma

**Affiliations:** 1 Division of Radiation Health, University of Arkansas for Medical Sciences, Little Rock, Arkansas, United States of America; 2 Department of Radiation Oncology, University of Arkansas for Medical Sciences, Little Rock, Arkansas, United States of America; 3 Department of Radiation Oncology, Moffitt Cancer Center and Research Institute, Tampa, Florida, United States of America; 4 Department of Pharmaceutical Sciences, University of Arkansas for Medical Sciences, Little Rock, Arkansas, United States of America; 5 Surgical Service, Central Arkansas Veterans Healthcare System, Little Rock, Arkansas, United States of America; Temple University, United States of America

## Abstract

Radiation-induced heart disease (RIHD) is a long-term side effect of radiotherapy of intrathoracic, chest wall and breast tumors when radiation fields encompass all or part of the heart. Previous studies have shown that pentoxifylline (PTX) in combination with α-tocopherol reduced manifestations of RIHD in rat models of local heart irradiation. The relative contribution of PTX and α-tocopherol to these beneficial effects are not known. This study examined the effects of PTX alone or in combination with tocotrienols, forms of vitamin E with potential potent radiation mitigation properties. Rats received localized X-irradiation of the heart with an image-guided irradiation technique. At 3 months after irradiation rats received oral treatment with vehicle, PTX, or PTX in combination with a tocotrienol-enriched formulation. At 6 months after irradiation, PTX-treated rats showed arrhythmia in 5 out of 14 animals. PTX alone or in combination with tocotrienols did not alter cardiac radiation fibrosis, left ventricular protein expression of the endothelial markers von Willebrand factor and neuregulin-1, or phosphorylation of the signal mediators Akt, Erk1/2, or PKCα. On the other hand, tocotrienols reduced cardiac numbers of mast cells and macrophages, but enhanced the expression of tissue factor. While this new rat model of localized heart irradiation does not support the use of PTX alone, the effects of tocotrienols on chronic manifestations of RIHD deserve further investigation.

## Introduction

Radiation-induced heart disease (RIHD) is a long-term side effect of radiotherapy of intrathoracic, chest wall and breast tumors when all or part of the heart was situated within the radiation fields. Several studies of survivors of Hodgkin’s disease and breast cancer report an increase in heart disease when examined 10-20 years after irradiation [1,2]. Manifestations of RIHD include accelerated atherosclerosis, pericardial and myocardial fibrosis, conduction abnormalities, and injury to cardiac valves [3]. Current radiotherapy protocols for cancer in the lung, esophagus or proximal stomach may deposit a significant radiation dose to the heart or parts thereof [4–6]. Advances in radiation therapy techniques are expected to enhance long-term survival in groups of patients that were previously difficult to treat, such as patients with lung cancer [7]. Nonetheless, pharmacological interventions specific for RIHD are not available.

Pentoxifylline is a non-selective inhibitor of cyclic nucleotide phosphodiesterases (PDE) that may induce increased intracellular levels of cyclic AMP (cAMP) and cyclic GMP (cGMP). The original clinical purpose of PTX was to improve erythrocyte plasticity [8]. However, PTX may also reduce fibroblast activity, improve endothelial function, and inhibit inflammation [9–12] and has beneficial effects in chronic radiation injury [13–15]. Studies by us and others have shown that PTX improved cardiac function and reduced adverse remodeling in rat models of localized heart irradiation when administered in combination with α-tocopherol, the most common analog of vitamin E [16,17]. Administrations that started one week before local heart irradiation or three months after irradiation were equally effective [16]. On the other hand, a rebound effect was observed when administration was discontinued during the chronic phase of RIHD [17]. The relative contribution of PTX and α-tocopherol to the beneficial effects of the combined administration is not known.

Vitamin E consists of at least eight analogs, four tocopherols and four tocotrienols. Several of these analogs have radioprotective properties [18,19]. Some studies suggest that tocotrienols accumulate in endothelial cells at higher rates compared to α-tocopherol [20], and induce a larger set of differentially expressed genes in endothelial cells [21]. In addition, γ- and δ-tocotrienols may mediate their protective effects in radiation injury in part by inhibition of 3-hydroxy-3-methyl-glutaryl-CoA reductase [18,22,23]. PTX enhanced the radioprotective properties of γ-tocotrienol in a combined administration before whole body irradiation [24].

This study aimed to identify the effects of PTX alone on cardiac radiation injury, and to investigate the effects of PTX when administered in combination with tocotrienols. For this purpose, a rat model of localized heart irradiation was used in which real-time imaging in combination with radiation treatment from multiple angles specifically targeted the heart but minimized exposure of surrounding tissues such as the lung and spinal cord.

## Materials and Methods

### Ethics statement

This study conformed to the Guide for the Care and Use of Laboratory Animals of the National Institutes of Health [25]. The protocol was approved by the University of Arkansas for Medical Sciences’ Institutional Animal Care and Use Committee (protocol # 3097).

### Animal model of local heart irradiation

Male Sprague-Dawley rats (Harlan Laboratories) were housed in the Division of Laboratory Animal Medicine on a 12:12 light-to-dark cycle with free access to food and water. Rats (220-260 g) were exposed to local heart irradiation with the Small Animal Conformal Radiation Therapy Device (SACRTD) as described before [26]. The SACRTD consists of a 225kVp X-ray tube (Isovolt Titan 225, General Electric) mounted on a gantry, a robotic arm positioning system (Viper^TM^ s650, Adept Technology), and a flat panel digital X-ray detector of 200 μm resolution (XRD 0820 CM3, PerkinElmer). A brass and aluminum collimating assembly was attached to the X-ray tube to produce a field of 19 mm diameter at the isocenter.

Dosimetry was performed as described before [26]. In short, the dose rate at the isocenter was measured using a pin-point ion chamber (PTW N301013, PTW) following the TG-61 protocol of the American Association of Physicists in Medicine [27]. In addition, dosimetry was performed with radiographic films (Gafchromic® EBT-2, Ashland Specialty Ingredients) that were calibrated with a Gamma Knife (Co-60) system (Elekta AB) and analyzed as described before [28]. To measure relative depth dose, 11 pieces of film were placed in between 11 slabs of solid water phantom each 5 mm thick. The film on the top of the phantom was kept at the isocenter, normal to the beam direction, and exposed to 5 Gy (225 kV, 13 mA).

Rats were anesthetized with 3% isoflurane and placed vertically in a cylindrical Plexiglas holder that was cut out such that no material was in between the radiation beam and the chest. The heart was irradiated with three 19 mm-diameter fields (anterior–posterior and two lateral fields) to 7 Gy each, using 225 kV, 13 mA, and 0.5 mm Cu-filtration, leading to 1.92 Gy/min at 1 cm tissue depth as measured with the dosimetry methods described above.

Before each 7-Gy exposure, an X-ray image was obtained with the 19 mm-diameter field using the built-in X-ray detector (70 kV, 5 mA, <1 cGy), to verify that the radiation field encompassed the heart. When necessary, the position of the rat was adjusted with the use of the robotic arm to place the heart in the middle of the radiation field.

### PTX and Tocomin SupraBio® administration

Previously, there was no difference in the effects of oral administration of PTX in combination with α-tocopherol when started one week before irradiation or when started at 3 months after irradiation [16]. Therefore, we started administration of PTX and tocotrienols at 3 months after local heart irradiation. PTX (Sigma-Aldrich) was added to our standard rodent chow (TD8640, Harlan-Teklad). Chow intake was monitored monthly to show that PTX intake was 95-110 mg/kg body weight/day. Small amounts of vitamin E were required to maintain the health of all animals. Hence, all rats received 11-13 mg α-tocopherol/kg bodyweight/day via the standard chow.

Tocomin SupraBio® (TSB) was kindly provided by Carotech. TSB is a tocotrienol-enriched mixture that contains 17% tocotrienols (8% γ-tocotrienol, 5% α-tocotrienol, 3% δ-tocotrienol, 1% β-tocotrienol) and 5% α-tocopherol in a self-emulsifying delivery system for enhanced oral absorption. TSB was administered at 250 mg/kg body weight/day via oral gavage. Hence, tocotrienols were administered at a dose of 43 mg/kg body weight/day. Together with the α-tocopherol in the chow, α-tocopherol intake was 15-17 mg/kg body weight/day.

Rats were randomly divided into four experimental groups: sham-irradiation (n=15) or local heart irradiation (n=15) with regular chow, local heart irradiation with PTX enriched chow (n=15), and local heart irradiation with PTX enriched chow in combination with TSB gavage (n=15). Rats were observed for 6 months after irradiation.

### Plasma levels of tocols

Blood samples from untreated rats and one rat that received TSB by oral gavage (250 mg/kg bodyweight) were collected into EDTA-coated tubes on ice. The tubes were centrifuged at 1,000g for 15 minutes at 4°C, and the supernatants were collected and centrifuged at 10,000g for 5 minutes. The plasma was collected, snap-frozen in liquid nitrogen and stored at -80°C until further analysis.

For the determination of tocols, 250 µl of stored plasma was thawed at room temperature, 2.5 µg of δ-tocopherol (internal standard) in 25 µl methanol, 500 µl of acetonitrile: tetrahydrofuran (3:2, v/v) were added, and the samples were mixed by vortexing for 5 min. The samples were centrifuged (12,800 g for 20 min at 10^°^C), and the supernatant was transferred to an amber vial and dried under nitrogen. To improve the recovery of the tocols, the pellet was suspended in 100 µl hexane, vortexed, centrifuged (12,800 g for 10 min at 10^°^C) and the supernatant was transferred to the same vial. The dried residues were analytically transferred to deactivated glass micro-inserts using methylene chloride, dried under nitrogen, and derivatized using *N*-methyl-N-TMS-trifluoroacetamide at 25^°^C. The derivatized samples were quantitated in triplicate by gas chromatography/mass spectrometry (5975 GC/MSD, Agilent) using single-ion monitoring and a 30-m HP-5MS column (0.250 mm, 0.25 µm). Samples were analyzed using helium as the carrier gas (head pressure of 27 psi), 1 µl split less injection. The injector temperature was 275°C, the column temperature was maintained at 220°C for 2 min followed by a gradient of 25°C/min to 300°C, and remained at that temperature for 10 min. The transfer line temperature was maintained at 285°C for 13.5 min followed by a gradient of 25°C/min to 300°C, and remained at that temperature for 10 min. The conditions were: electron impact, source temperature 230°C, quadrupole temperature 150°C, and ionization voltage 70 eV. This method does not distinguish between β- and γ-tocotrienol.

### Echocardiography

Echocardiography was performed as described before [29], using a Vevo 2100 imaging system (VisualSonics) with the MS250 transducer (13-24 MHz). Animals were anesthetized with 2% isoflurane and hair was removed from the chest with clippers followed by a depilatory cream. The rats were placed on a warmed platform that recorded ECGs from the paws. ECGs were used to detect bradycardia (heart rate <250 bpm) and arrhythmia. Short axis M-mode recordings at the mid left ventricular level were used to obtain left ventricular anterior wall (LVAW), posterior wall (LVPW), inner diameter (LVID), ejection fraction (EF), fractional shortening (FS), stroke volume, cardiac output, and heart rate.

### Langendorff perfused rat heart preparations

Langendorff studies were performed as described before [30]. In short, animals were anesthetized with 3% isoflurane, and hearts were isolated and immediately perfused via the aorta with an oxygenated Krebs-Henseleit solution (37°C) at a flow rate of 10 mL/g heart/min. The ventricles were paced with platinum contact electrodes to a heart rate of 250 beats/min. Atria were removed and a fluid-filled balloon connected to a pressure transducer (model PT300, Grass Technologies) was placed in the left ventricle to measure diastolic and systolic pressures at balloon volumes between 80 µL and 300 µL. Coronary pressure was monitored continuously with a second pressure transducer (model PT300, Grass Technologies).

After Langendorff studies the hearts were weighed and processed for histology and immunohistochemistry.

### Histology and immunohistochemistry

Hearts were fixed in methanol Carnoy’s solution (60% methanol, 30% chloroform, 10% acetic acid) and embedded in paraffin. Histology and immunohistochemistry were performed as described before [29]. In short, for determination of collagen deposition and areas of myocardial degeneration, sections were incubated in Sirius Red (American MasterTech) supplemented with Fast Green (Fisher Scientific). For determination of mast cell numbers, sections were incubated in 0.5% Toluidine Blue in 0.5 N HCl, followed by 0.7 N HCl.

After blocking of endogenous peroxidase with 1% H_2_O_2_ in methanol and blocking of non-specific antibody binding with 10% normal donkey or goat serum in 3% dry powdered milk and 0.2% BSA, sections were incubated overnight with rabbit anti-von Willebrand factor (vWF, 1:800, Dako), mouse anti-CD68 (1:100, Abcam), or rabbit anti-Tissue Factor (TF, 1:200, Santa Cruz), followed by goat anti-rabbit IgG (1:400, Vector Laboratories) or donkey anti-mouse IgG (1:400, Fitzgerald Industries) and an avidin-biotin-peroxidase complex (Vector Laboratories). Bound antibodies were visualized with 0.5 mg/mL 3,3-diaminobenzidine tetrahydrochloride (Sigma-Aldrich).

Sirius Red stain and vWf immunostainings were analyzed with an Axioskop transmitted light microscope (Carl Zeiss) equipped with a chilled color camera (Leica). Areas of myocardial degeneration, areas positive for Sirius Red, and areas positive for vWf were quantified with dedicated image analysis software (Image-Pro Plus 5.1, Media Cybernetics).

### Western-Blots

Western-Blots were performed as described before [29]. In short, animals were anesthetized with 3% isoflurane, and left ventricular tissue samples were isolated, snap-frozen and subsequently stored at -80°C. Left ventricular tissue was homogenized in RIPA buffer with inhibitor cocktails of proteases and phosphatases (Sigma Aldrich). A total of 50 µg protein was prepared in Laemmli buffer containing β-mercaptoethanol (1: 20 vol/vol) and boiled for 2-3 minutes. Protein samples were separated in polyacrylamide gels (Any kD^TM^ Mini-Protean® or 4-20% gradient, Bio-Rad) and transferred to PVDF membranes. After blocking in 5% dry powdered milk, membranes were incubated with rabbit anti-phosphorylated PKCα (1:15,000 Upstate Biotechnology), mouse anti total PKCα (1:15,000 BD Biosciences), rabbit anti-phospho-Akt, rabbit anti-phospho-Erk1/2, rabbit anti-Erk1/2, (all three 1: 10,000 Cell Signaling Technology), rabbit anti neuregulin-1 (Nrg-1, 1:1,000 Santa Cruz), or rabbit anti-TF (1:2,000 Santa Cruz). Protein loading was corrected with mouse anti-GAPDH (1:20,000 Santa Cruz). Primary antibodies were followed by HRP-conjugated goat anti-mouse (Jackson ImmunoResearch) at a a dilution of 1:20,000 for GAPDH and 1:5,000 for all other mouse primary antibodies, or mouse anti-rabbit (Cell Signaling Technology) at a dilution of 1:7,500 for Nrg-1 and TF, and 1:4,000 for all other rabbit primary antibodies. Antibody binding was visualized with ECL^TM^ Plus Western Blotting Detection reagent (General Electric Healthcare Life Sciences). Protein bands on scanned films were quantified with ImageJ.

### RNA isolation and real-time PCR

Left ventricular gene expression was assessed with real-time PCR as described before [29]. In short, animals were anesthetized with 3% isoflurane, and left ventricular tissue samples were isolated, snap-frozen and subsequently stored at -80°C. Left ventricular samples were homogenized in Ultraspec^TM^ RNA reagent (Biotecx Laboratories) and treated with DNAse (RQ-DNAse I, Promega). cDNA was synthesized using the High Capacity cDNA Archive Kit^TM^ (Applied Biosystems). Real-time PCR was performed with the 7500 Fast Real-Time PCR System and the following pre-designed TaqMan Gene Expression Assays^TM^: Nrg-1 (Rn01482165_m1), thrombomodulin (TM, Rn00582226_s1), tumor necrosis factor-α (TNF-α, Rn01525859_g1), Interleukin-1β (IL-1β, Rn00580432_m1), Interleukin-6 (IL-6, Rn01410330_m1), and PKCα (Rn01496145_m1) (all Applied Biosystems). Relative mRNA levels were calculated with the ΔΔCt method, using 18S rRNA as normalizer.

### Statistical analysis

Data were evaluated with two-way ANOVA, followed by Newman-Keuls individual comparisons. Correlations between parameters were evaluated with linear regression. All statistical analysis was performed with the software package NCSS 8 (NCSS). The criterion for significance was a p<0.05.

## Results

### Oral gavage with TSB caused increased plasma levels of tocotrienols

To verify that rats absorbed vitamin E analogs after oral gavage with TSB, we measured plasma concentrations of α-tocopherol and tocotrienols of rats that were on regular chow and received TSB gavage and compared these results with rats that were on regular chow. Plasma α-tocopherol of rats on regular chow was 2.8 ± 1.2 μg/ml (n=3), and tocotrienols were undetectable. After gavage with TSB, plasma concentration of α-tocopherol was at least 4.8 μg/ml, while the concentrations of δ-tocotrienol and the combined γ- and β-tocotrienol reached 1.3 μg/ml and 0.8 μg/ml, respectively (Figure S1).

### The experimental procedures did not alter heart or body weight

Heart and body weights were not altered by radiation or any of the treatments (data not shown), and animals showed no other signs of physical distress. One animal in the PTX group had to be sacrificed before the endpoint of the experiment, due to a skin lesion that occurred outside the radiation field.

### TSB reduced cardiac numbers of mast cells and CD68-positive cells

**Figure 1:**
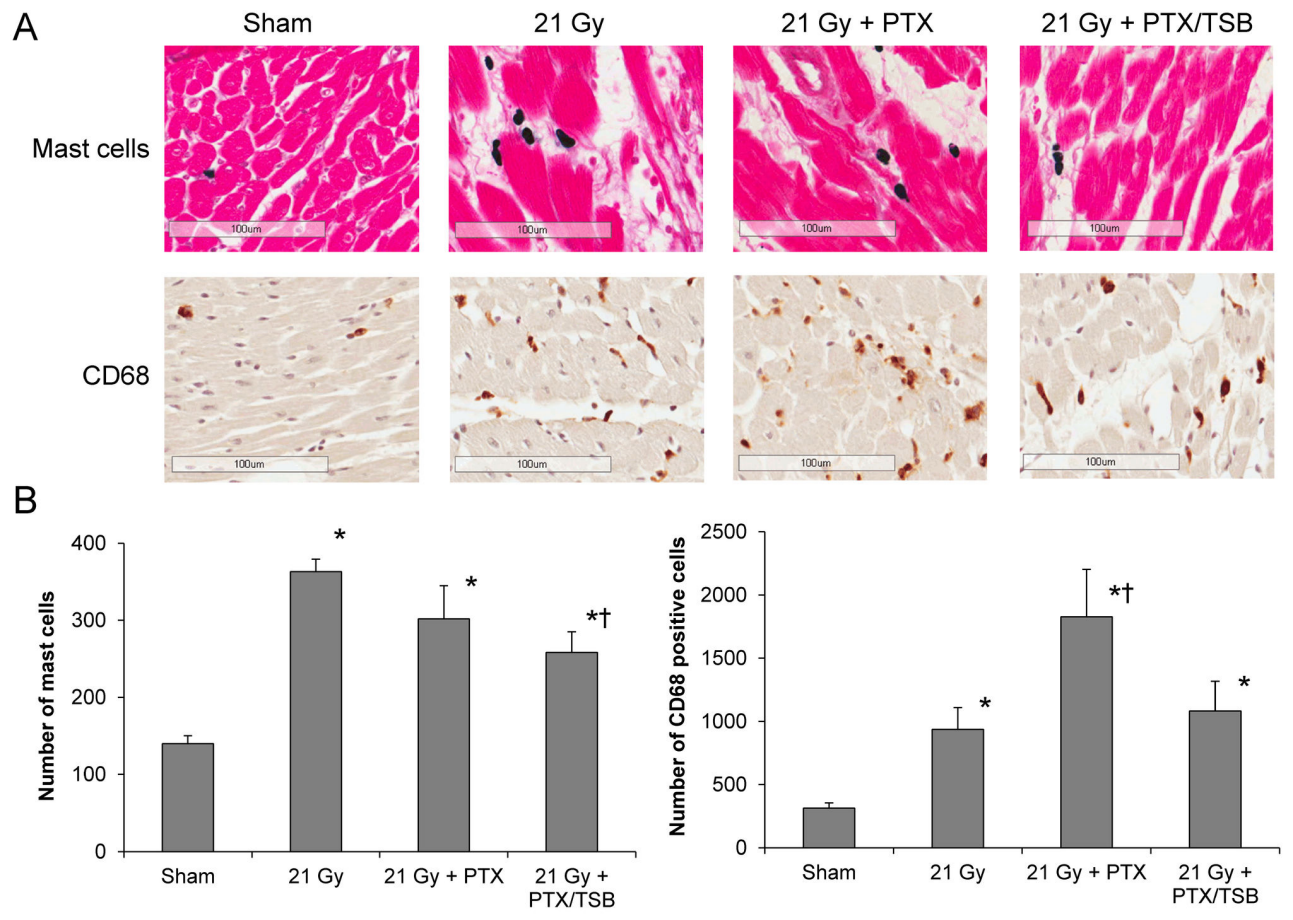
Cardiac inflammatory cell numbers at 6 months after irradiation. Radiation caused an increase in mast cell numbers and CD68 positive cells, as shown by representative micrographs (A) and total cell counts (B). TSB reduced the effects of radiation on cardiac mast cell number. PTX enhanced the effects of radiation on the number of CD68 positive cells. Average ± SEM, n=8-9. *Significant difference with sham-irradiation (p<0.05), ^†^ Significant difference with 21 Gy (p<0.05). Scale bar: 100 μm.

Radiation is known to cause increased numbers of mast cells and macrophages in the heart. Because PTX and tocotrienols have anti-inflammatory effects, we hypothesized that they may reduce radiation-induced inflammatory infiltration in the heart. As expected, radiation-induced myocardial injury coincided with increased cardiac numbers of macrophages and mast cells (Figure 1). PTX significantly enhanced the number of CD68-positive cells in irradiated animals, while addition of TSB to the treatment significantly reduced both the number of CD68 positive cells and the number of mast cells (Figure 1).

Despite changes in the numbers of CD68-positive cells, left ventricular gene expression of the macrophage-related cytokines TNF-α, IL-1β and IL-6 did not change (data not shown).

### PTX and TSB modified some endothelial cell properties

Endothelial dysfunction may play an important role in RIHD. Since both PTX and tocotrienols are known to have positive effects on endothelial cell function, and tocotrienols seem to accumulate to relatively high concentrations in endothelial cells, we studied the expression of the endothelial markers vWf, Nrg-1, TM, TF, and eNOS.

**Figure 2:**
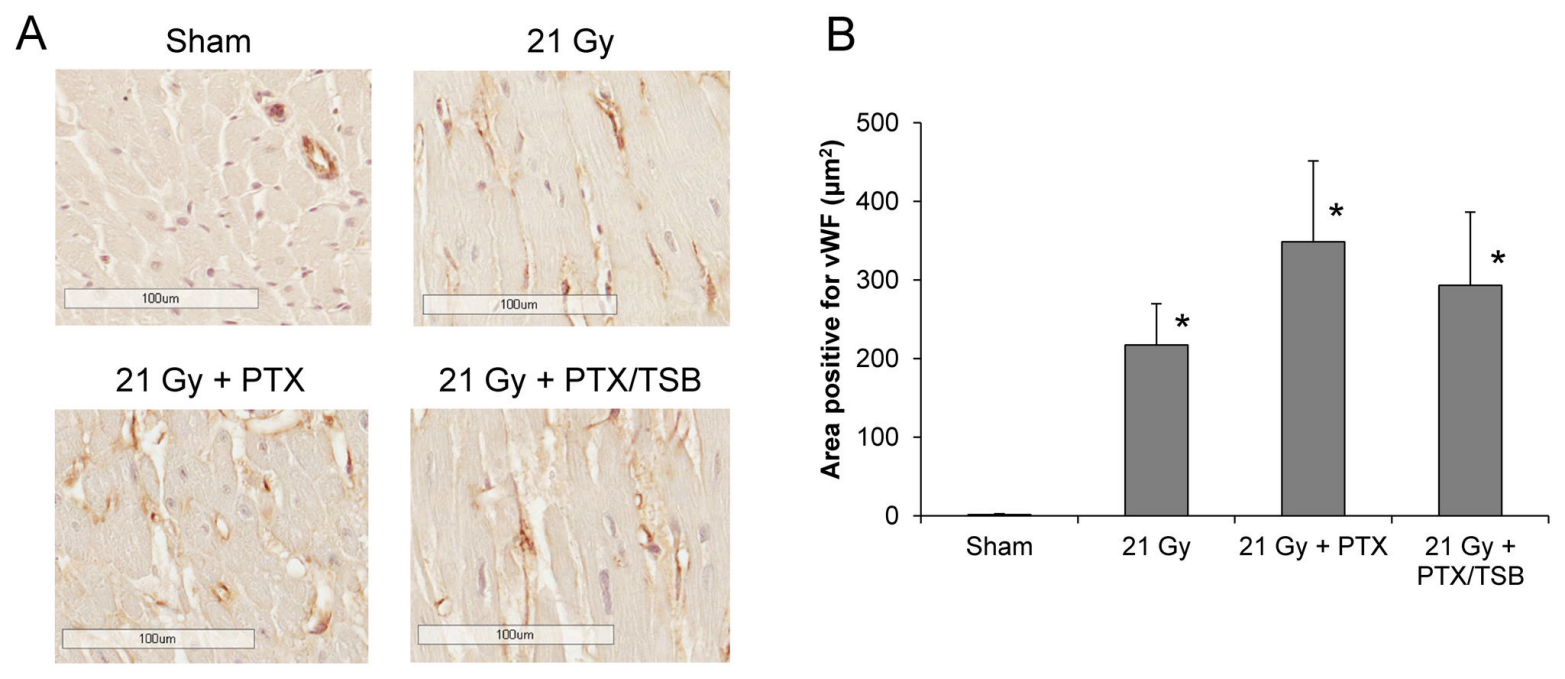
Cardiac expression of vWf at 6 months after irradiation. In sham-irradiated hearts, vWF immunoreactivity was only found on the endothelium of larger arteries, while in irradiated hearts vWF was also found in endothelial cells of capillaries and arterioles, and in some hearts in the extracellular matrix (A). Areas immunoreactive for vWf were not altered by PTX or TSB (B). Average ± SEM, n=8-9. *Significant difference with sham-irradiation (p<0.05). Scale bar: 100 μm.

VWf is a prothrombotic and profibrogenic mediator that is induced in injured endothelial cells. In sham-irradiated hearts, vWF immunoreactivity was only found on the endothelium of larger arteries, while in irradiated hearts vWF was also found in endothelial cells of capillaries and arterioles, and in some hearts in the extracellular matrix. These immunoreactive areas of vWF strongly correlated with the area of interstitial fibrosis (R^2^=0.46, p<0.001). PTX and TSB did not significantly alter the radiation-induced increases in vWF area (Figure 2).

**Figure 3:**
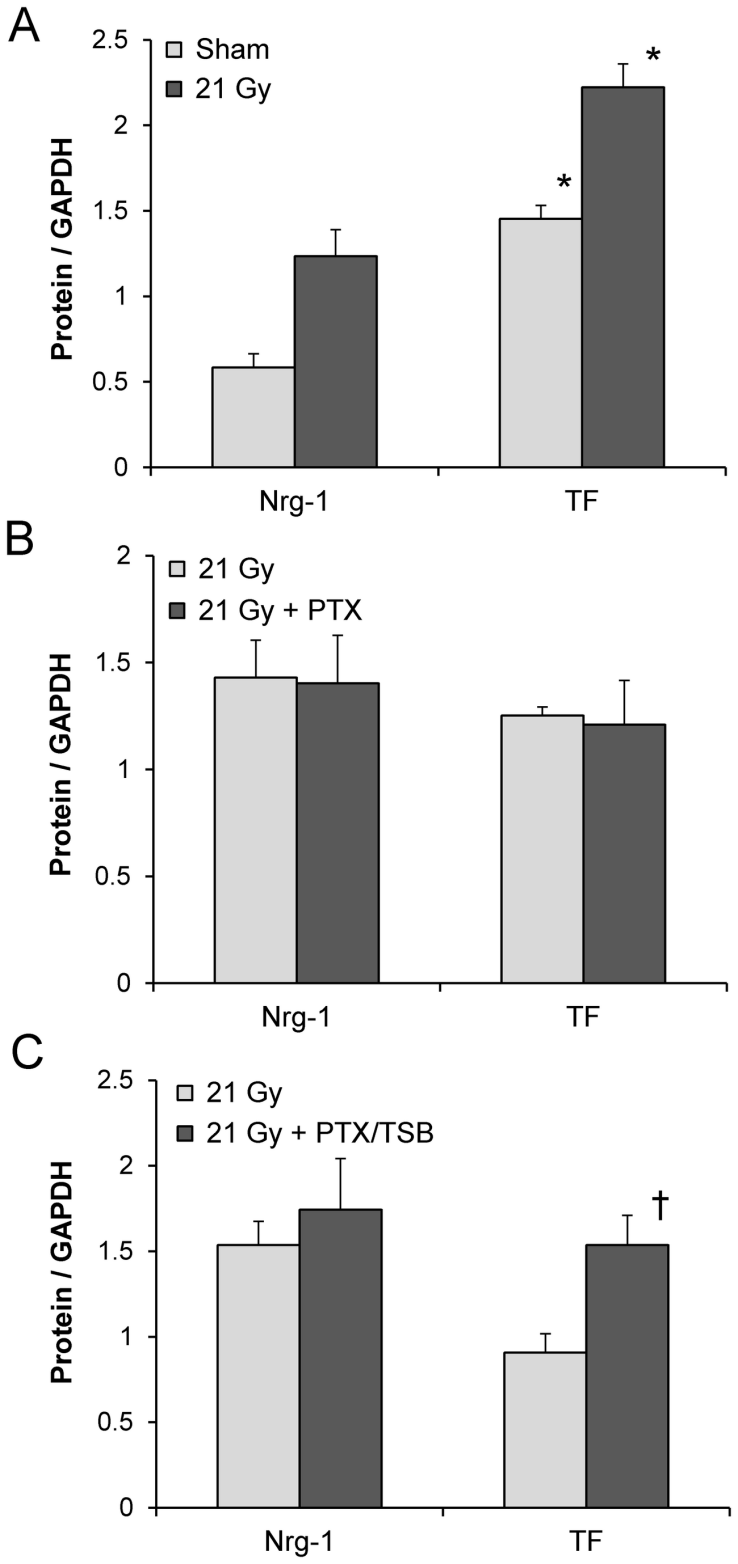
Left ventricular protein levels of Nrg-1 and TF at 6 months after irradiation. Radiation caused a significant increase in Nrg-1 and TF expression (A). PTX did not alter Nrg-1 or TF (B), while PTX in combination with TSB enhanced TF expression (C). Average ± SEM, n=4-5. *Significant difference with sham-irradiation (p<0.05), ^†^ Significant difference with 21 Gy (p<0.05). Scans of the Western-Blot films are shown in Figure S2.

Nrg-1 is required for survival and function of cardiomyocytes. Microvascular endothelial cells are the main source of Nrg-1 in the heart. Radiation caused a significant increase in gene and protein expression of Nrg-1. While PTX in combination with TSB enhanced Nrg-1 mRNA (Table 1), changes in Nrg-1 protein were not significant (Figure 3, Figure S2).

**Table 1:** Left ventricular mRNA levels of TM, Nrg-1, and PKCα at 6 **months**.

**Experimental group**	**TM**	**Nrg-1**	**PKCα**
Sham	1.04 ± 0.14	1.58 ± 0.69	1.01 ± 0.08
21 Gy	1.38 ± 0.11	3.79 ± 0.50*	2.13 ± 0.13*
21 Gy + PTX	1.84 ± 0.20^†^	3.50 ± 0.40	1.70 ± 0.20*
21 Gy + PTX/TSB	1.76 ± 0.23^†^	5.61 ± 0.48*^†^	1.78 ± 0.09*

Relative mRNA levels were measured with real-time PCR at 6 months after local heart irradiation (average ± SEM, n 5).

* Significant difference with sham-irradiation (p<0.05), † Significant difference with 21 Gy (p<0.05).

TM is an antithrombotic endothelial cell surface mediator that is reduced in certain models of normal tissue radiation injury. While real-time PCR revealed a significant induction of TM mRNA by PTX (Table 1), TM protein levels were near the detection limits of Western-Blot and immunohistochemistry with no apparent difference between experimental groups (data not shown).

TF is a proinflammatory mediator that becomes expressed on injured endothelial cells in certain situations. In all experimental groups, a small subset of capillaries and arterioles stained positive for TF (Figure S3). Western-Blots revealed an increase in TF expression with PTX in combination with TSB (Figure 3, Figure S2).

### Radiation reduced left ventricular ratios of phosphorylated to total Akt, Erk1/2 and PKCα

**Figure 4:**
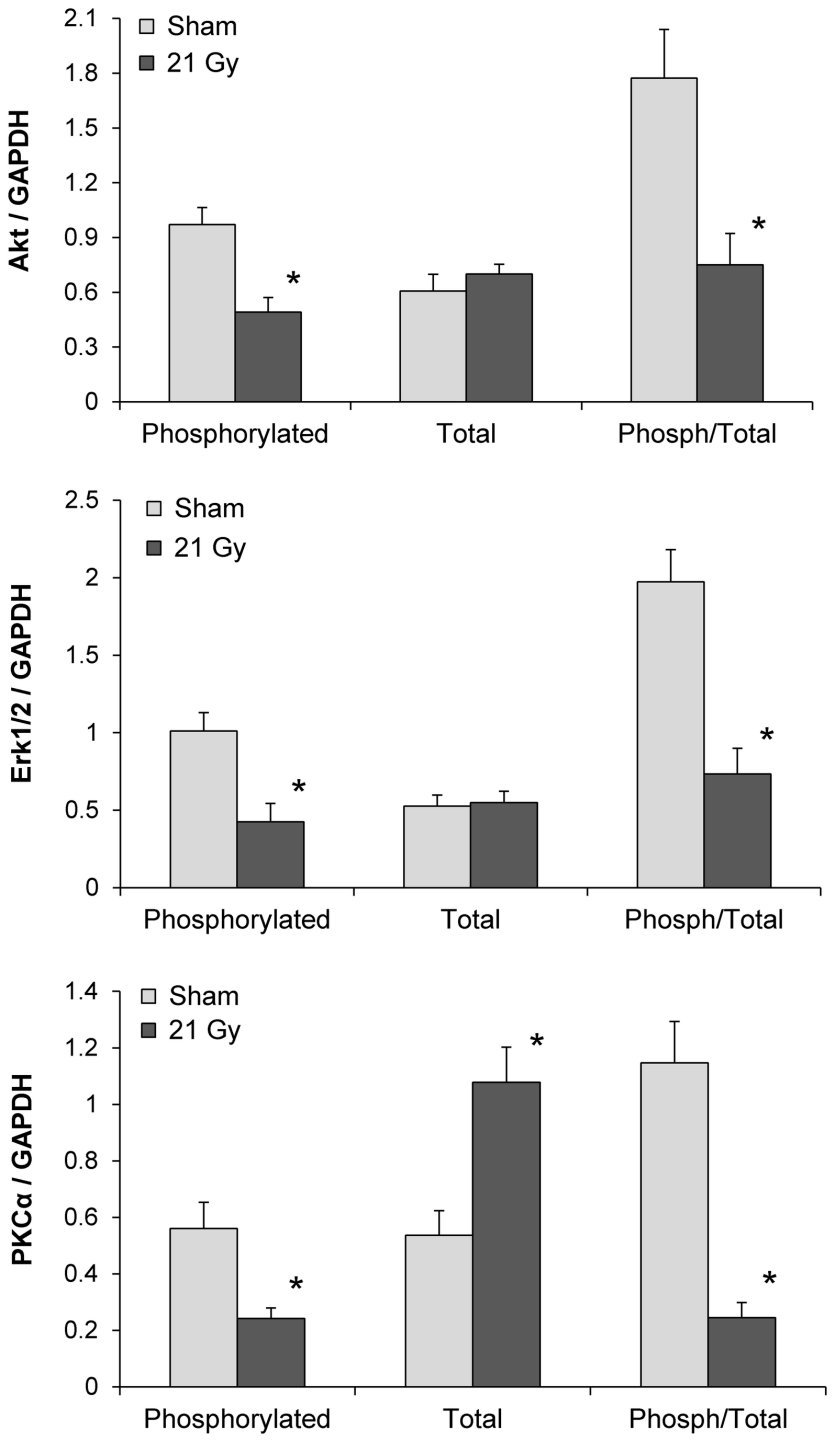
Left ventricular protein levels of total and phosphorylated Akt, Erk1/2, and PKCα at 6 months. Radiation caused a significant reduction in the phosphorylated form of all three signaling molecules. Scans of the Western-blot films are shown in Figure S4. Average ± SEM, n=6. *Significant difference with sham-irradiation (p<0.05).

Intracellular signaling pathways that involve Erk1/2, Akt and PKCα are known for their role in cardiac physiology and disease. Local heart irradiation caused increased left ventricular levels of PKCα transcripts (Table 1) and protein, while phosphorylation of Erk1/2, Akt, and PKCα was reduced (Figure 4, Figure S4).

**Figure 5:**
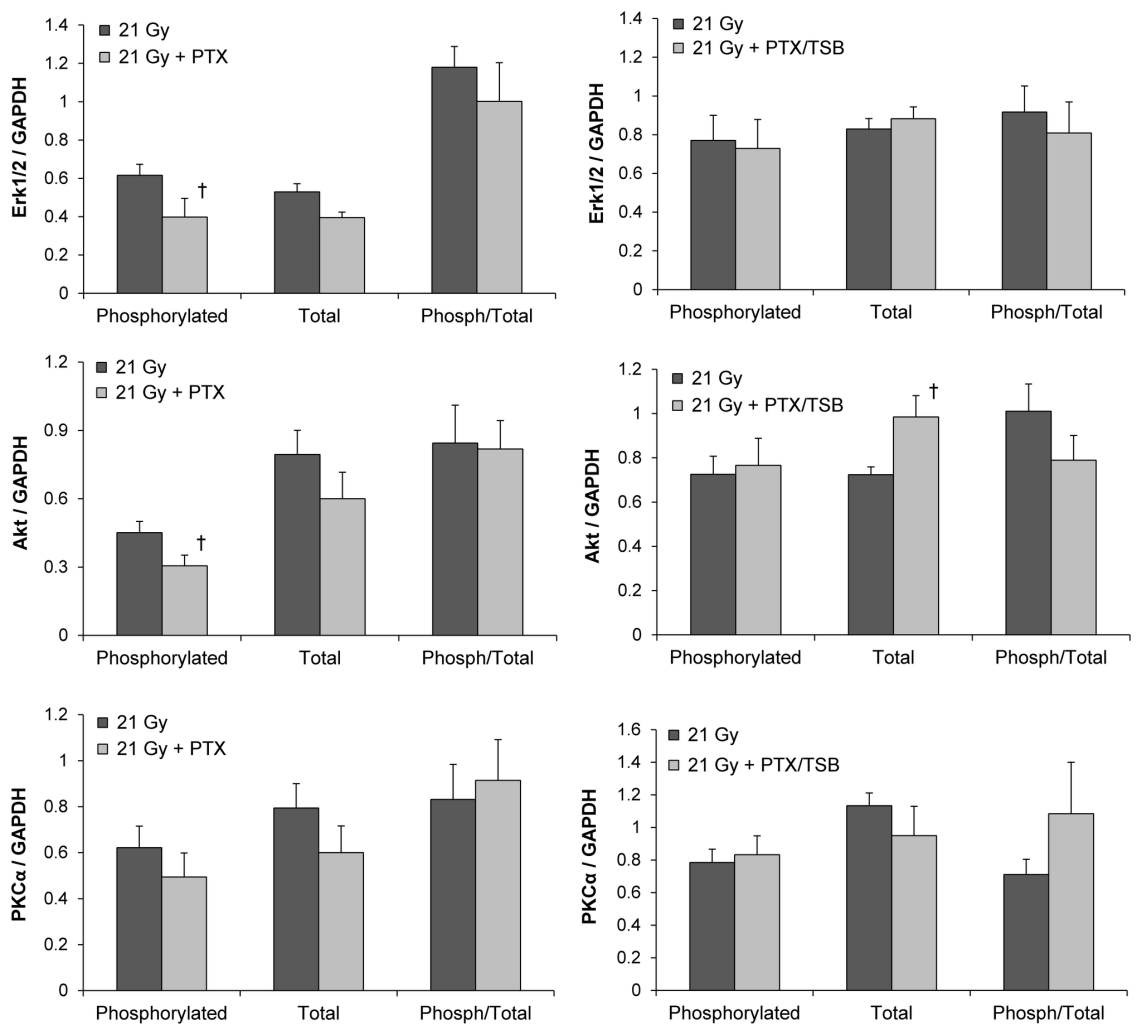
Effects of PTX and TSB on left ventricular Akt, Erk1/2, and PKCα in irradiated animals. PTX alone reduced the levels of phosphorylated Erk1/2 and phosphorylated Akt, but did not significantly change the ratio of phosphorylated to total Erk1/2 or Akt. PTX in combination with TSB increased protein levels of total Akt, but did not change the ratio of phosphorylated to total Akt. PTX and TSB had no effect on PKCα. Scans of the Western-blot films are shown in Figure S5. Average ± SEM, n=6. ^†^Significant difference with 21 Gy (p<0.05).

We hypothesized that PTX and TSB may alter radiation-induced cardiac injury by modifying some or all of these signaling molecules. PTX further reduced the levels of phosphorylated Erk1/2 and phosphorylated Akt in irradiated animals when administered alone, but not when administered in combination with TSB. Addition of TSB caused an increase in expression of total Akt. However, the ratios of phosphorylated to total Akt, Erk1/2 and PKCα were not significantly altered by the two treatments (Figure 5, Figure S5).

### PTX worsened some of the radiation-induced changes in cardiac function

Radiation-induced inflammation, endothelial dysfunction, and changes in Erk1/2, Akt and PKCα signaling may all contribute to functional and histopathological manifestations of RIHD. To determine the time course of the effects of radiation on cardiac function, and the effects of PTX and TSB on radiation-induced cardiac injury, *in vivo* cardiac function was examined with echocardiography at 3 and 6 months after irradiation. Results are shown in Table S1. Radiation induced an increase in LVAW and LVPW thickness in systole. In addition, both time points showed a reduction in heart rate, which was statistically significant at 3 months. Hence, at this time point an increased stroke volume was required to maintain the same cardiac output.

Treatment with PTX and TSB started immediately after the 3-months echocardiography session and lasted until 6 months after irradiation. In our rat model of local heart irradiation, we typically find only one in 10-20 animals with bradycardia and/or arrhythmia. Accordingly, in the current study one rat out of 15 animals on regular chow showed bradycardia (heart rate <250 bpm) and arrhythmia at 6 months after irradiation. PTX treatment caused a significant increase in LVID in diastole, suggestive of dilated cardiomyopathy, and also increased the incidence of bradycardia and arrhythmia (Table S1). There was no apparent relationship between the P waves and QRS complexes in the ECG recordings of the animals with arrhythmia, suggesting that these animals had a third-degree heart block (Figure S6). Heart function was particularly affected in this sub-group of animals: EF increased to 77.6%, FS increased to 48.5%, and stroke volume to 334.4 μl. Nevertheless, cardiac output (calculated as stroke volume x heart rate), was reduced to 55.1 ml/min. Addition of TSB did not significantly correct the effects of PTX on echocardiographic parameters (Table S1).

### None of the treatments changed ex vivo Langendorff-perfused rat heart parameters

In addition to *in vivo* measures of cardiac function, *ex vivo* cardiac function parameters were also obtained from Langendorff perfused hearts at 6 months after irradiation. Hearts were isolated and *ex vivo* perfused in a Langendorff apparatus to measure left ventricular and coronary pressures. These Langendorff parameters were not significantly altered in irradiated hearts or in hearts from animals treated with PTX or PTX combined with TSB (data not shown).

### Effects of PTX and TSB on histopathological changes were not significant

**Figure 6:**
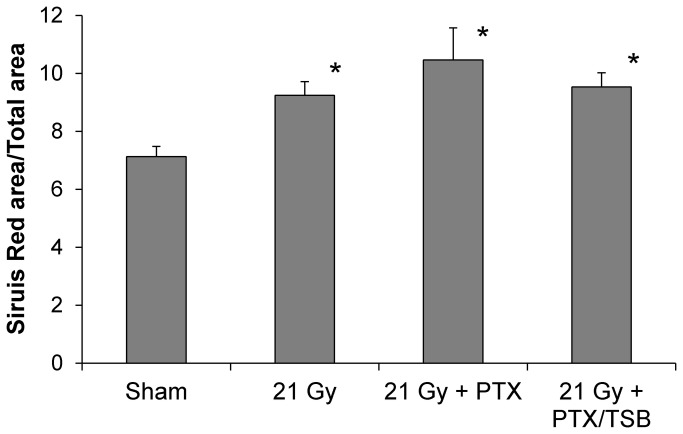
Analysis of collagen deposition at 6 months after irradiation. Radiation caused increases in interstitial collagen, as measured by interstitial areas staining positive with Sirius Red. Interstitial collagen areas were not altered by PTX or TSB. Average ± SEM, n=8-9. *Significant difference with sham-irradiation (p<0.05).

Local heart irradiation is known to cause myocardial degeneration, and perivascular and interstitial fibrosis. We hypothesized that PTX and TSB would reduce these radiation-induced histopathological changes. We therefore analyzed areas of myocardial degeneration and quantified myocardial collagen deposition. One out of 9 sham-irradiated hearts had an area of myocardial degeneration of more than 200 µm, compared to 7 out of 9 irradiated hearts. Radiation-induced interstitial fibrosis (Figure 6) and myocardial degeneration were not significantly altered by PTX alone (5 out of 8 hearts showed degeneration in an area >200 μm) or PTX and TSB (3 out of 9 hearts showed degeneration in an area >200 μm).

## Discussion

The current study used a model of image-guided irradiation to precisely target the heart and reduce exposure of the lungs and spinal cord in the rat, to examine the effects of post-radiation administration of PTX alone and PTX combined with a tocotrienol-enriched formulation. Our previous method of rat heart irradiation involved parallel opposed fields (AP, PA 1:1) given as a single dose or given in five fractions of 9 Gy [16,30]. While histopathological changes, including myocardial degeneration, fibrosis, and increased numbers of inflammatory cells are consistent with the results of single dose or fractionated irradiation with the previous model, some of the outcomes of the current protocol are different. While previously increased diastolic pressures were observed in Langendorff preparations of hearts isolated at 6 months after irradiation, diastolic pressures no longer change in the current radiation model. Lung injury can cause left ventricular diastolic dysfunction [31]. Hence, combined heart and lung exposure to radiation leads to more severe diastolic dysfunction [32]. Although our two radiation models were not compared simultaneously, existing evidence from other studies make it plausible that reduced radiation exposure of the lungs in the current study may explain why changes in *ex vivo* diastolic pressure no longer occur.

Previous studies have shown that PTX improved cardiac function and reduced adverse remodeling when administered in combination with α-tocopherol after local heart irradiation in rats [16,17]. In our previous studies, PTX was administered at 100 mg/kg bodyweight/day in combination with α-tocopherol at 40 mg/kg body weight/day [16]. Here, apart from the small amount of α-tocopherol in the rat chow, PTX is tested in the absence of higher doses of α-tocopherol. Similar to our previous studies, PTX caused some changes in echocardiographic parameters, with no change in the number of cardiac mast cells. On the other hand, PTX did not significantly alter myocardial radiation fibrosis. Based on the considerations of reduced radiation exposure of the lung in this study, it may be possible that PTX mostly inhibits adverse myocardial remodeling that is indirect to radiation injury in the lung.

The dosage of PTX was well within what is considered the safe range for PTX in the rat. Nonetheless, PTX caused bradycardia and arrhythmia in 5 out of 14 irradiated animals. The family of PDE enzymes aids in calcium homeostasis and the regulation of heart rhythm. Hence, PDE inhibitors may make the heart more susceptible to arrhythmia [33,34]. Interestingly, this phenomenon was not observed in our previous experiments with PTX [16]. The more targeted radiation exposure of the heart in the present study has revealed that prolonged treatment with PTX may make the irradiated heart more vulnerable to bradycardia and arrhythmia.

This is the first study to investigate the effects of tocotrienols in chronic radiation injury in the heart. Vitamin E consists of at least eight tocol analogs, four tocopherols and four tocotrienols, of which α-tocopherol is most commonly found in vitamin E supplements and also most commonly studied. Because tocols are fat-soluble, their absorption and plasma transport depends on dietary fat content. This study administered TSB, a tocotrienol-enriched formulation in a self-emulsifying delivery system for oral absorption. We found that this formulation produced substantial peak plasma levels, comparable to those in human subjects that receive oral tocotrienols [35].

Endothelial dysfunction contributes to a pro-coagulant, pro-inflammatory, and pro-fibrotic environment in normal tissue radiation injury [36,37]. Hence, increased deposition of vWF, a marker of endothelial dysfunction, is seen in the irradiated heart [38,39]. Although PTX and tocotrienols may protect against endothelial dysfunction [40,41], PTX and TSB did not significantly change vWF immunoreactive areas. Nrg-1 is a ligand in the epidermal growth factor pathway that is expressed by cardiac microvascular endothelial cells. Nrg-1 is required for the survival of cardiomyocytes [42]. Hence, Nrg-1 plays a protective role in animal models of heart disease [43,44]. We previously found that left ventricular Nrg-1 is down-regulated during the first 10 weeks after local heart irradiation in rats, followed by an up-regulation at 6 months and later time points [45]. In the current study, TSB enhanced radiation-induced increases in left ventricular mRNA but not protein. We also examined the endothelial marker TM. TM is expressed on the luminal surface of endothelial cells where it influences coagulation and inflammation [46,47]. Reduced expression of TM is an indication of endothelial dysfunction after exposure to ionizing radiation [48]. However, no alterations in TM expression were seen in late cardiac radiation injury in the mouse [39]. Endothelial TM expression is controlled by cAMP, and PTX is known to upregulate its expression [11,12]. While PTX treatment enhanced TM mRNA levels, we could not provide evidence for an increase in TM protein. Lastly, TF is a prothrombotic endothelial mediator that is overexpressed after exposure to ionizing radiation in certain tissues [49]. We found that an increase in left ventricular protein levels of TF was enhanced by PTX in combination with TSB. Further examination is required to determine the exact effects of PTX and TSB on endothelial cell function in the irradiated heart.

Radiation-induced myocardial injury is accompanied by increased cardiac numbers of mast cells and macrophages [27,38]. Increases in mast cells and macrophages at 6 months after irradiation did not coincide with changes in left ventricular mRNA levels of TNF-α, IL-1β, or IL-6. These results suggest that, instead of inducing a late pro-inflammatory environment, radiation may cause the recruitment of inflammatory cells to injured myocardium. Indeed, many iron-containing macrophages can be found in the irradiated heart [38,50]. On the other hand, macrophages may enhance cardiac fibrosis [51] and may therefore contribute to late radiation fibrosis in the heart. TSB reduced the numbers of both mast cells and macrophages. Indeed, various analogs of vitamin E can alter mast cell proliferation, survival and degranulation [52]. In our study, maximum tocotrienol plasma levels were obtained at 2-4 hours after administration. Hence, an administration schedule of at least twice daily oral treatment may enhance the effects of TSB on radiation injury.

Intracellular signaling pathways that involve Akt, Erk1/2 and PKCα are known for their role in cardiac physiology and disease. Both Akt and Erk1/2 may have favorable effects in the heart with their pro-survival and pro-angiogenic properties and promotion of cardiac contractility [53]. Hence, reduced activity of Akt and Erk1/2 is related to chronic adverse remodeling in the heart [54,55]. Increased PKCα activity, on the other hand, may reduce cardiac contractility and enhance heart failure [56]. Consistent with chronic structural changes in the irradiated heart, phosphorylation of all three signaling mediators was reduced. Long-term effects of PTX or tocotrienols on Akt, Erk1/2, or PKCα in the heart have not yet been reported. In our rat model of RIHD, PTX with or without TSB did not seem to alter these signaling pathways.

In conclusion, oral administration of PTX alone from 3 months to 6 months after irradiation did not significantly alter adverse myocardial remodeling or intracellular signaling, but instead had an adverse effect on cardiac rhythm after local heart irradiation. While addition of a tocotrienol-enriched oral formulation could not correct the adverse effects of PTX on cardiac function, it reduced myocardial inflammatory infiltration. Hence, potential mitigation of RIHD with tocotrienols, alone or in combination with other potential radiation modifiers, deserves further examination.

## Supporting Information

Figure S1 Plasma concentrations of α-tocopherol and tocotrienols after oral gavage with TSB. TSB was administered at a dose of 250 mg/kg body weight.
(TIF)

Figure S2 Scans of Western-Blots to examine endothelial markers. The effects of radiation, PTX, and PTX in combination with TSB on left ventricular expression of Nrg-1 and TF were examined at 6 months after local heart irradiation.
(TIF)

Figure S3 Representative micrographs of the immunohistochemical analysis of TF at 6 months after local heart irradiation. Scale bar: 100 μm.
(TIF)

Figure S4 Scans of Western-Blots to examine Akt, Erk1/2, and PKCα after local heart irradiation. Left ventricular total and phosphorylated Akt, Erk1/2, and PKCα were examined at 6 months after local heart irradiation.
(TIF)

Figure S5 Scans of Western-Blots to examine the effects of PTX or PTX in combination with TSB on Akt, Erk1/2, and PKCα. The effects of PTX, and PTX in combination with TSB on left ventricular total and phosphorylated Akt, Erk1/2, and PKCα were examined at 6 months after local heart irradiation.
(TIF)

Figure S6 Example ECG traces at 6 months after sham-irradiation, and of bradycardia and arrhythmia after irradiation. Bradycardia and arrhythmia occurred in 1 out 15 irradiated rats treated with vehicle, 5 out 14 irradiated rats treated with PTX, and 6 out of 15 irradiated rats treated with PTX and TSB.
(TIF)

Table S1 Echocardiographic M-mode analysis parameters at 3 months and 6 months after local heart irradiation. Treatment with PTX and TSB started immediately after the 3-months echocardiography session and lasted until 6 months after irradiation. Average ± SEM, n=14-44.
(PDF)
