# Supplementary figures and images for: Effects of Late Administration of Pentoxifylline and Tocotrienols in an Image-Guided Rat Model of Localized Heart Irradiation
Source: PLoS One. 2013 Jul 22;8(7):e68762. doi: 10.1371/journal.pone.0068762 (PMC3718790; doi:10.1371/journal.pone.0068762)

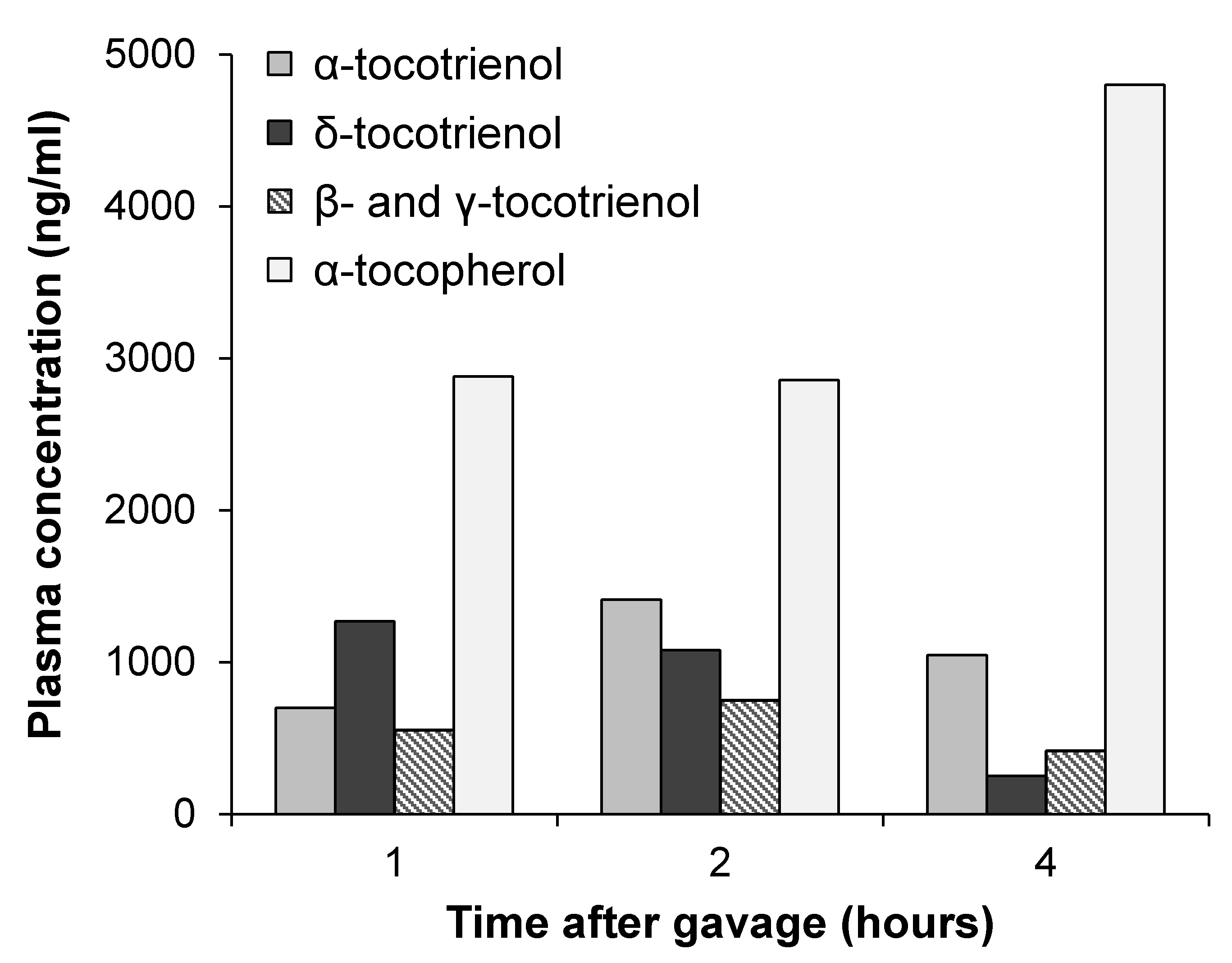

Supplement: Figure S1 — TSB was administered at a dose of 250 mg/kg body weight. (TIF) [file pone.0068762.s001.tif]

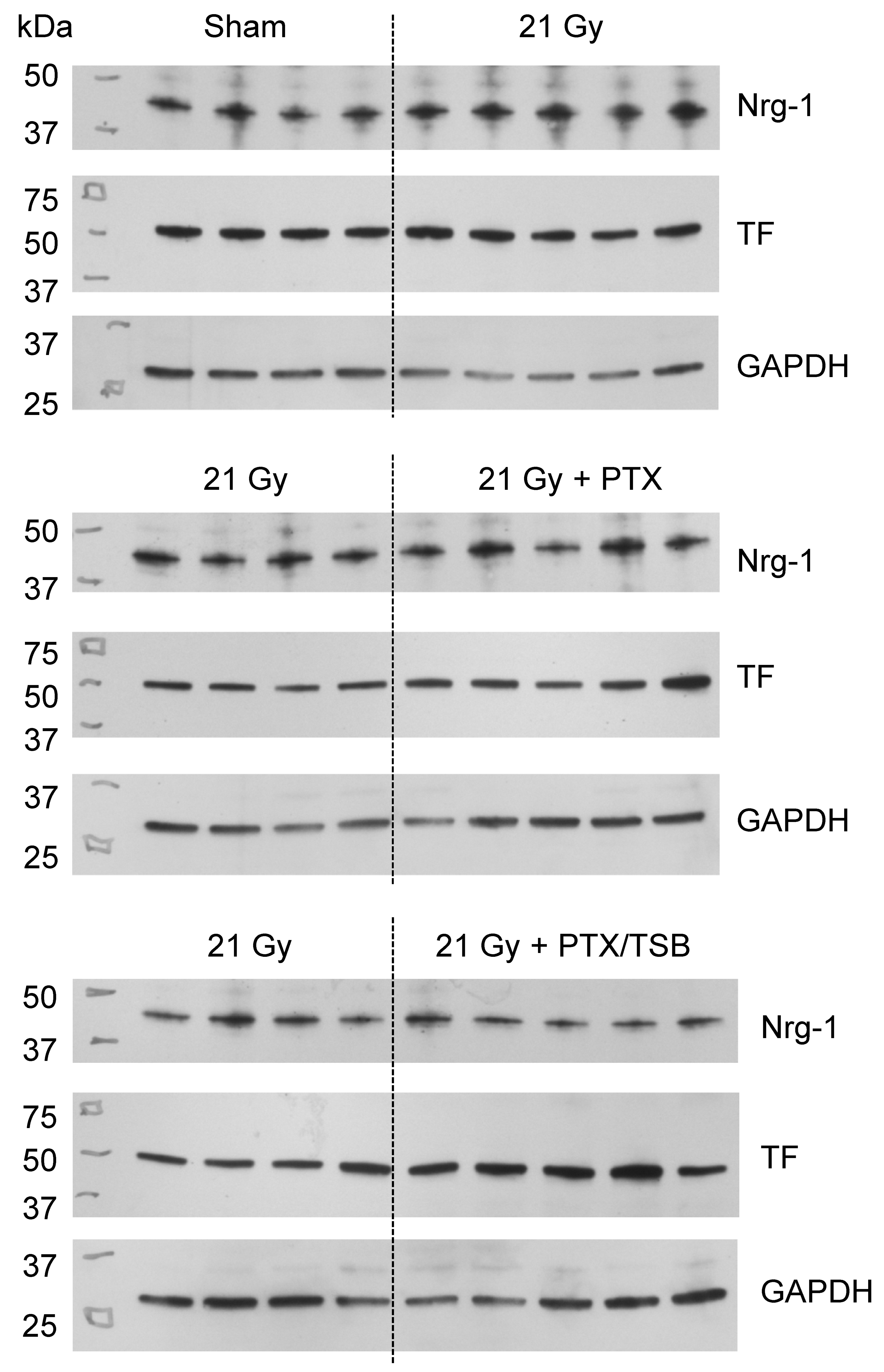

Supplement: Figure S2 — The effects of radiation, PTX, and PTX in combination with TSB on left ventricular expression of Nrg-1 and TF were examined at 6 months after local heart irradiation. (TIF) [file pone.0068762.s002.tif]

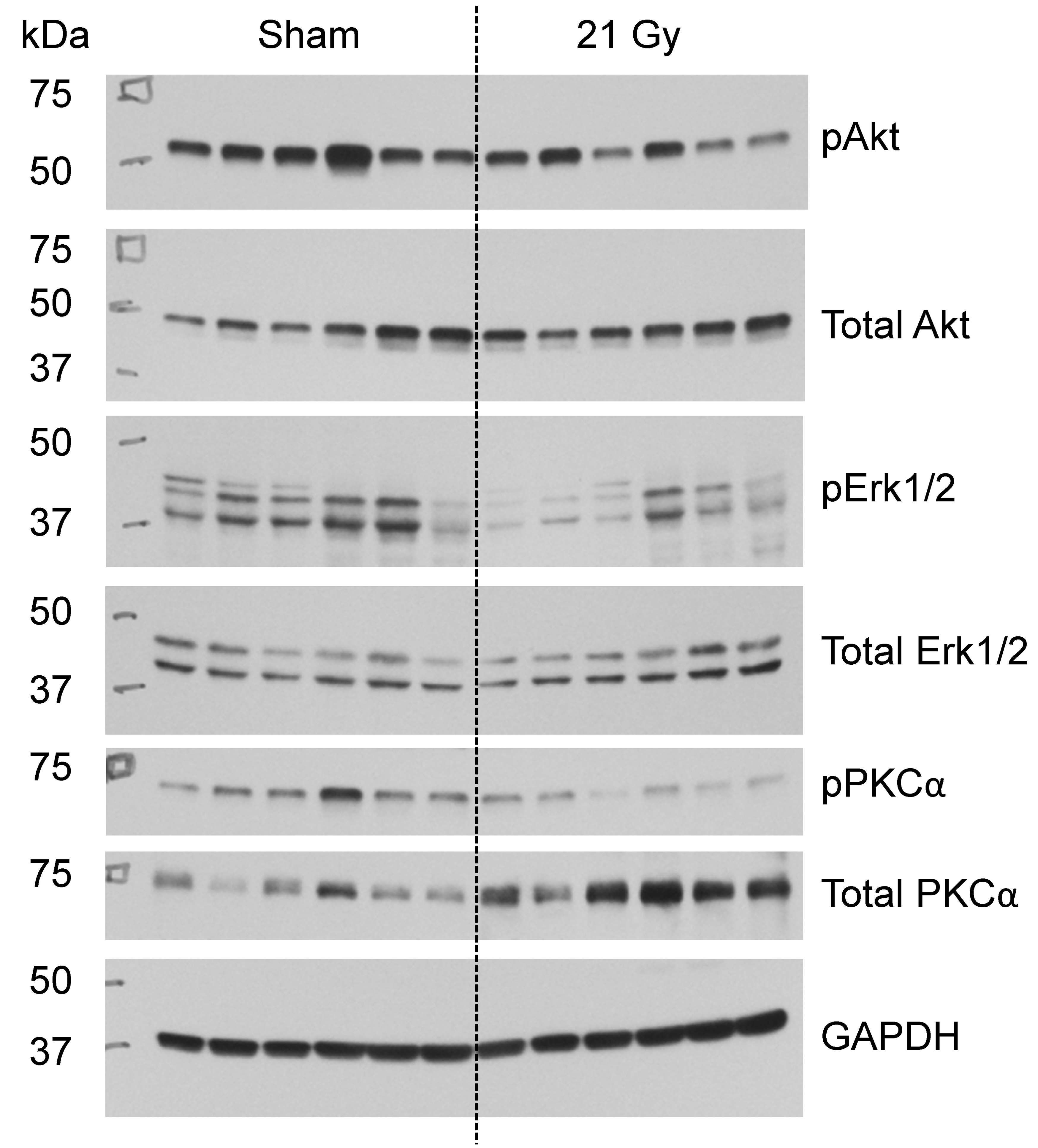

Supplement: Figure S4 — Left ventricular total and phosphorylated Akt, Erk1/2, and PKCα were examined at 6 months after local heart irradiation. (TIF) [file pone.0068762.s004.tif]

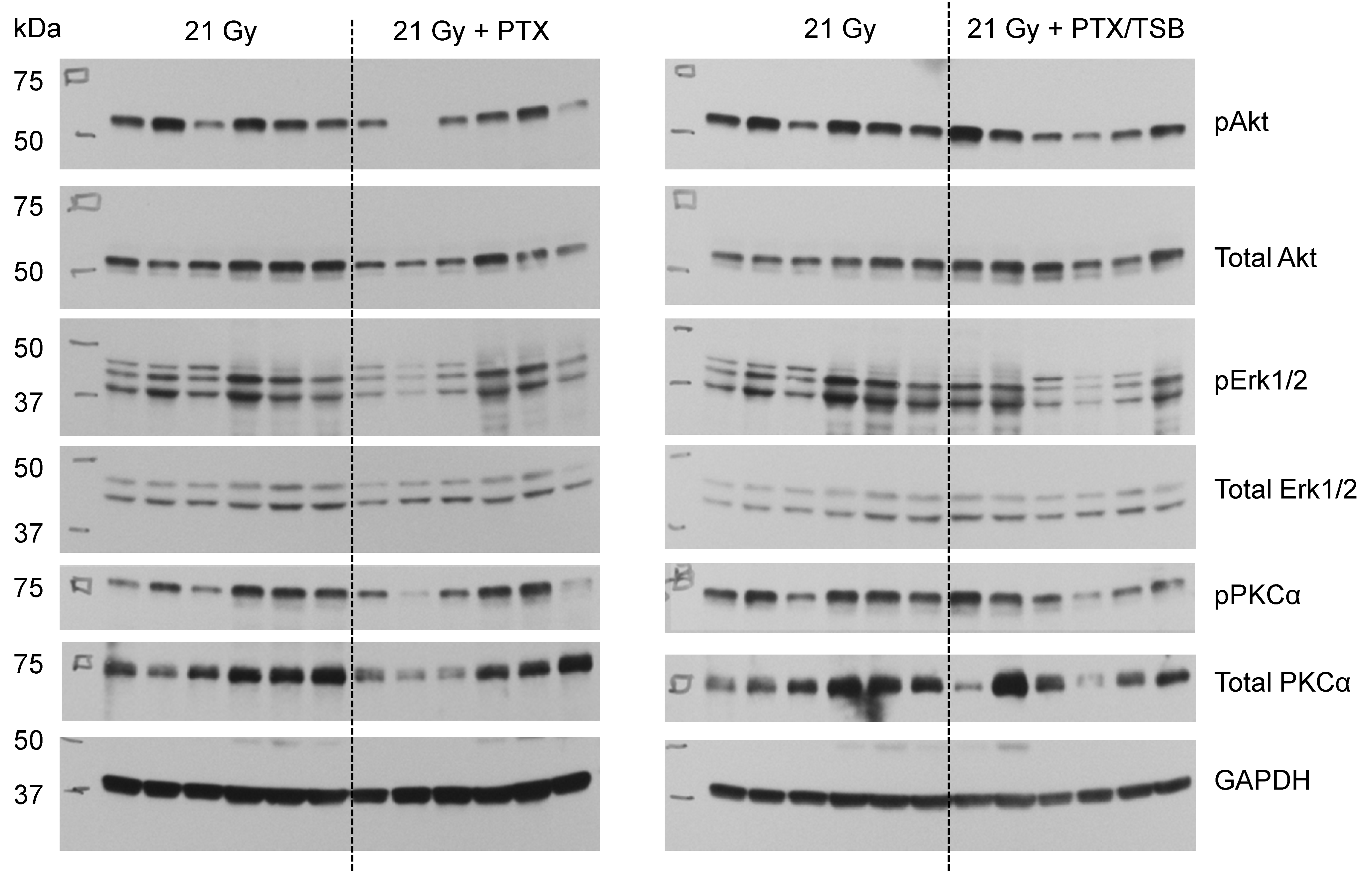

Supplement: Figure S5 — The effects of PTX, and PTX in combination with TSB on left ventricular total and phosphorylated Akt, Erk1/2, and PKCα were examined at 6 months after local heart irradiation. (TIF) [file pone.0068762.s005.tif]

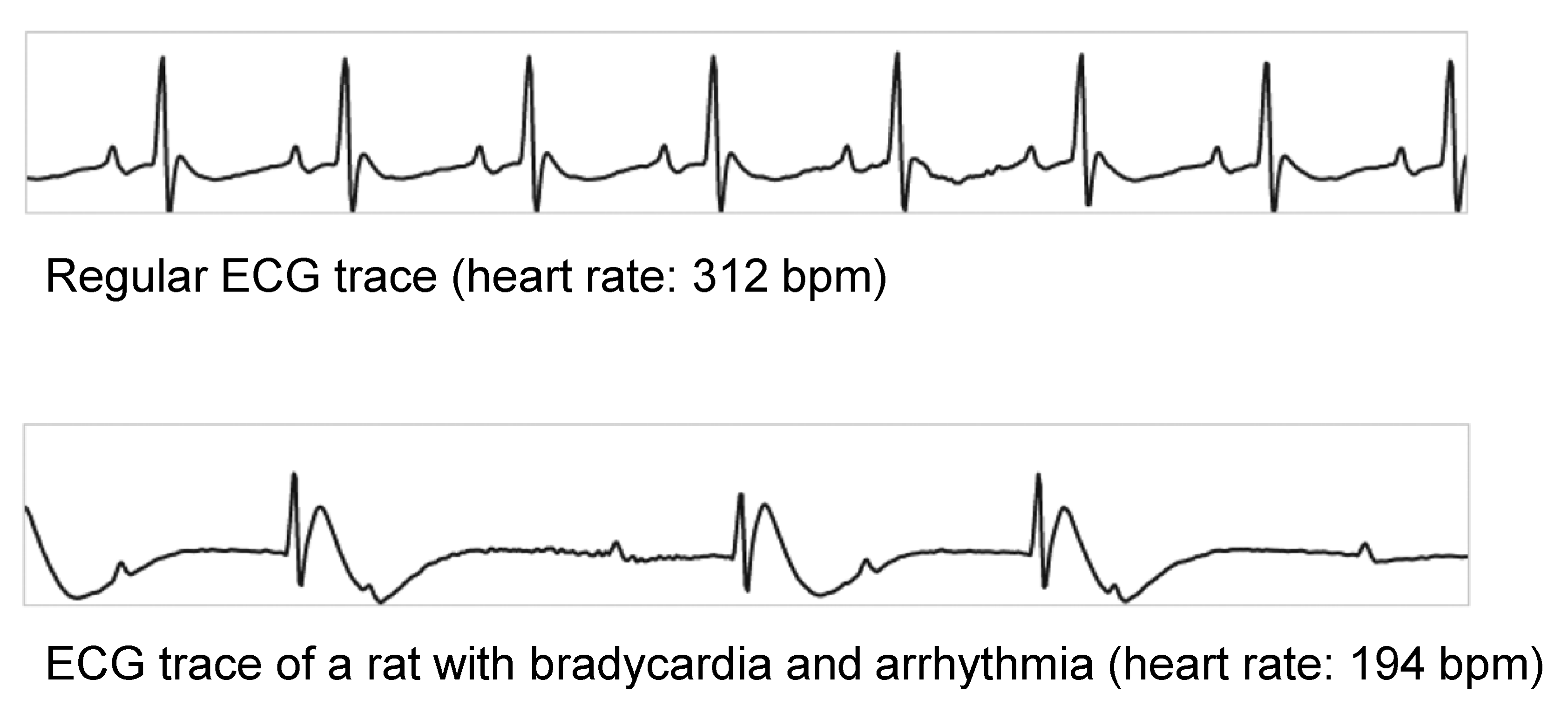

Supplement: Figure S6 — Bradycardia and arrhythmia occurred in 1 out 15 irradiated rats treated with vehicle, 5 out 14 irradiated rats treated with PTX, and 6 out of 15 irradiated rats treated with PTX and TSB. (TIF) [file pone.0068762.s006.tif]
