# Supplementary material for: Effects of Late Administration of Pentoxifylline and Tocotrienols in an Image-Guided Rat Model of Localized Heart Irradiation
Source: PLoS One. 2013 Jul 22;8(7):e68762. doi: 10.1371/journal.pone.0068762 (PMC3718790; doi:10.1371/journal.pone.0068762)
Supplement: Table S1 — Treatment with PTX and TSB started immediately after the 3-months echocardiography session and lasted until 6 months after irradiation. Average ± SEM, n=14-44. (PDF) [file pone.0068762.s007.pdf]

Table S1. Echocardiographic M-mode analysis parameters at 3 months and 6 months after local heart irradiation.

	3 months after irradiation		6 months after irradiation			
	Sham	21 Gy	Sham	21 Gy	21 Gy + PTX	21 Gy + PTX/TSB
LVAW in diastole (mm)	1.71 ± 0.06	1.80 ± 0.03	1.80 ± 0.05	1.84 ± 0.04	1.68 ± 0.05†	1.81 ± 0.05
LVAW in systole (mm)	2.69 ± 0.09	2.99 ± 0.05*	2.76 ± 0.10	3.03 ± 0.11	2.97 ± 0.12	3.04 ± 0.10*
LVPW in diastole (mm)	1.72 ± 0.04	1.82 ± 0.03	1.80 ± 0.05	1.91 ± 0.05	1.84 ± 0.06	1.81 ± 0.05
LVPW in systole (mm)	2.67 ± 0.07	2.88 ± 0.05*	2.68 ± 0.07	2.90 ± 0.07	2.76 ± 0.08	2.82 ± 0.08
LVID in diastole (mm)	7.45 ± 0.13	7.66 ± 0.06	7.80 ± 0.15	8.00 ± 0.10	8.46 ± 0.09*†	8.49 ± 0.09*†
LVID in systole (mm)	4.59 ± 0.14	4.45 ± 0.09	5.07 ± 0.19	4.69 ± 0.20	4.92 ± 0.19	4.92 ± 0.17
EF (%)	71.47 ± 1.50	74.42 ± 0.97	68.65 ± 1.65	73.77 ± 1.94	71.87 ± 2.48	73.17 ± 1.89
FS (%)	42.39 ± 1.25	45.20 ± 0.92	40.16 ± 1.33	44.91 ± 1.78	43.27 ± 2.22	44.42 ± 1.65
Stroke volume (µl)	215.53 ± 5.43	238.8 ± 4.37*	238.10 ± 5.11	265.04 ± 6.94	288.94 ± 13.39*	299.59 ± 11.68*†
Heart rate (bpm)	325.9 ± 4.03	307.62 ± 3.03*	307.66 ± 7.13	279.66 ± 10.79	258.62 ± 17.18	248.14 ± 21.44*
Cardiac output (ml/min)	70.11 ± 1.52	73.30 ± 1.31	73.22 ± 3.97	73.34 ± 2.99	71.87 ± 2.48	72.51 ± 5.14
Incidence of bradycardia and arrhythmia	0 out of 15	0 out of 44	0 out of 15	1 out of 15	5 out of 14	6 out of 15

*Significant difference with sham-irradiation ($p<0.05$), †Significant difference with 21 Gy ($p<0.05$).
